# Calycosin induces autophagy and apoptosis *via* Sestrin2/AMPK/mTOR in human papillary thyroid cancer cells

**DOI:** 10.3389/fphar.2022.1056687

**Published:** 2022-12-16

**Authors:** Na Qu, Junsheng Qu, Na Huang, Kexin Zhang, Tongtong Ye, Junfeng Shi, Bing Chen, Chengxia Kan, Jingwen Zhang, Fang Han, Ningning Hou, Xiaodong Sun, Ruiyan Pan

**Affiliations:** ^1^ Department of Endocrinology and Metabolism, Affiliated Hospital of Weifang Medical University, Weifang, China; ^2^ Clinical Research Center, Affiliated Hospital of Weifang Medical University, Weifang, China; ^3^ Department of Pathology, Affiliated Hospital of Weifang Medical University, Weifang, China; ^4^ School of Pharmacy, Weifang Medical University, Weifang, China

**Keywords:** calycosin, thyroid cancer, small molecules, therapy, autophagy, Sestrin2

## Abstract

Calycosin, one of small molecules derived from astragalus, has anti-tumor effects in various tumors. However, the effects of calycosin on papillary thyroid cancer (PTC) remain unclear. This study aimed to explore the anti-tumor ability of calycosin on human PTC and its potential mechanisms. The B-CPAP cells were treated with calycosin, then cell proliferation, apoptosis and invasiveness were measured by CCK8 assay, flow cytometry, wound healing and transwell invasion assay, respectively. The cells were also performed by whole transcriptome microarray bioinformatics analysis. Apoptosis and autophagy-related markers or proteins were measured by qRT-PCR or western blot. Sestrin2-mediated AMPK/mTOR pathways were determined by western blot. We found that calycosin inhibited migration and invasion of B-CPAP cells and induced apoptosis (Bax/Bcl-2) and autophagy (LC3II/I, Beclin1) of B-CPAP cells. Differential expressed genes were screened between the calycosin-treated cells and control (524 genes upregulated and 328 genes downregulated). The pathway enrichment suggested that the role of calycosin in B-CPAP cells is closely related to apoptosis-related genes and p70S6 Kinase. Transmission electron microscopy found an increase in autophagosomes in calycosin-treated cells. Sestrin2 in human PTC tissues and B-CPAP cells was lower than in normal thyroid tissues and cells. And the pharmacological effects of calycosin in PTC cells were related to Sestrin2 activation, increased p-AMPK and inhibited p-mTOR and p-p70S6Kinase; these alterations were reversed when silencing Sestrin2. In conclusion, calycosin has an inhibitory effect on PTC *via* promoting apoptosis and autophagy through the Sestrin2/AMPK/mTOR pathway.

## Introduction

Thyroid cancer is the most common endocrine tumor and head and neck cancer with increasing incidence (Siegel et al., 2021). Papillary thyroid cancer (PTC) is the type with the largest proportion, accounting for about 85%–90% of all thyroid cancers (Wells Jr, 2016). PTC is a pathological type of thyroid cancer with good differentiation and high survival rates and is usually inert and curable with a 5-year survival rate >95% (Schlumberger, 1998). However, in some cases, about 30% of PTCs still dedifferentiate and become aggressive, leading to tumor recurrence and distant metastases (Beierwaltes et al., 1982). Surgical resection remains the primary treatment for PTC, supplemented by radioiodine therapy, thyroid-stimulating hormone suppression therapy, local radiotherapy, and targeted drug therapy (Filetti et al., 2019). Selecting potentially useful drugs is undoubtedly feasible for patients with advanced thyroid cancer or who lose the chance of operation (Ancker et al., 2019). Thus, developing clinically effective drugs based on pathogenesis is essential.

Autophagy, the intracellular degradation process of damaged proteins and organelles, is an alternative way of programmed cell death different from apoptosis (Gudipaty et al., 2018). Autophagy maintains cellular homeostasis and regulates cell growth and death (Li et al., 2021). Autophagy plays a dual role in tumors, promoting or suppressing *via* regulating apoptosis (Levy et al., 2017). Studies have reported that activating autophagy could inhibit the progression of PTC by inducing apoptosis (Zhang et al., 2015). Moreover, autophagy activators could enhance the chemosensitivity and therapeutic response during PTC treatment (Lin et al., 2010).

Sestrin (SESN) is a highly conserved protein encoded by genes whose cell expression is up-regulated when exposed to DNA damage, oxidative stress and hypoxia (Qu et al., 2021). It includes three isoforms: SESN1, SESN2, and SESN3(Lee et al., 2013). Among them, SESN2 is a downstream target gene of p53, encoded by hypoxia-inducible gene 95 (Maiuri et al., 2009). Mainly expressed in mammals, SESN2 can resist oxidative stress damage, regulate cellular metabolism, and regulate autophagy and apoptosis (Sun et al., 2020). Current studies have shown that Sestrin2 initiates cellular autophagy through the AMP-activated protein kinase (AMPK)/mammalian target of rapamycin complex 1 (mTORC1) pathway, i.e.; SESN2 activates the AMPK pathway and inhibits downstream mTORC1-P70S6K to promote autophagy (Peng et al., 2014; Wolfson et al., 2016). Similar to autophagy, the role of SESN2 in tumors is paradoxical (Qu et al., 2021). Some studies have found that SESN2 is a tumor suppressor gene, such as neuroblastoma (Ambrosio et al., 2017), bladder cancer (Liang et al., 2016), colorectal cancer (Wei et al., 2015), and endometrial cancer (Shin et al., 2020). However, other studies confirm that it is a cancer-promoting gene, such as lung cancer (Byun et al., 2017), osteosarcoma (Tang et al., 2021), and melanoma cells (Zhao et al., 2014). However, the research of Sestrin2 in thyroid cancer is still not fully elucidated.

Calycosin (C_16_H_12_O_5_), one of the main components of astragalus (Gong et al., 2016), has been proven to have anti-inflammatory (Dong et al., 2018), antioxidant (Zhai et al., 2020), neuroprotective (Yang et al., 2019), antitumor (Tian et al., 2017) and vascular protective effects (Han et al., 2018). Its biological effects may be related to its flavonoids. Current research has confirmed that calycosin has anti-tumor properties and can exert its anti-tumor effects through multiple pathways and targets (Wang et al., 2019). Calycosin induced apoptosis *via* PI3K/AKT/mTOR signaling pathway in osteosarcoma cells (Sun et al., 2018). Moreover, calycosin can induce colorectal cancer cells (HT29) through the SIRT1/AMPK pathway-mediated autophagy (El-Kott et al., 2019). However, the effect of calycosin on autophagy and apoptosis of thyroid cancer is unknown. Thus, this study examined whether calycosin could have antitumor effects and the molecular mechanisms underlying SESN2-mediated autophagy in PTC.

## Materials and methods

### Cell culture

The human papillary thyroid (B-CPAP) cancer cells and the human normal thyroid cells (Nthy-ori 3-1) were from the National Collection of Authenticated Cell Cultures (Shanghai, China). The medium was fresh RPMI1640 (Gibco, China) with 10% fetal bovine serum and 1% penicillin and streptomycin (Invitrogen, Carlsbad, CA, United States).

### Proliferation assay

Calycosin powder dissolved in dimethyl sulfoxide was diluted with RPMI1640 to the appropriate concentration. The cancer cells with 5 × 10^3^ cells/well and cultured with calycosin at various concentrations (0, 25, 50, and 100 μM) for 24 h. Absorbance was measured at 450 nm after using the CCK8 Assay Kit (Sparkjade, China).

### Colony formation

800 cells/well of B-CPAP were plated on a six-well plate with calycosin (100 μM) or control (0 μM) for 12 days; cell clones were fixed with methanol for 15 min. Subsequently, the cell clones were stained for 30 min by adding giemsa stain solution (Solarbio, Beijing, China) and photographed.

### Wound healing assay

B-CPAP cells were cultured in six-well plates for 24 h to allow cells to confluence; wounds were created through the center line using a pipette tip. Photographs were taken to measure the distance between the wound edges. The culture was then continued for 24 h with calycosin, photographs were taken, and the distance between wound edges was measured by ImageJ software.

### Transwell assay

The B-CPAP cells were added into transwell membranes (Corning, NY, United States) at 1 × 10^4^ cells/well in medium and calycosin. The lower chamber of the 24-well plate contains conventional cell culture medium. After 24 h, the upper chambers were fixed with methanol, stained for 45 min by giemsa stain solution, and photographed with a microscope (Olympus, Tokyo, Japan).

### Flow cytometry analysis

After being treated with calycosin (100 μM) or control (0 μM) for 24 h, the cells were fixed with 70% ethanol, stained by Annexin V-FITC Apoptosis Detection Kit (BD, United States), and analyzed in FACS with CellQuest software version 3.3.

### Transmission electron microscopy

The B-CPAP cells were inoculated to a density of about 80%, treated with calycosin or control group for 24 h, then washed with PBS, fixed with 2% glutaraldehyde, and then photographed and imaged by transmission electron microscopy (HT7800/HT7700, Hitachi, Japan).

### Cell transfection

Cell transfection was performed using lipofectamine 3,000 (Invitrogen, United States). siRNA directed against SESN2 (si-SESN2: sense (5′-3′)CCG​AAG​AAU​GUA​CAA​CCU​CUU, antisense (5′-3′) AAG​AGG​UUG​UAC​AUU​CUU​CGG) and negative control siRNA (siNC) were from Qingke Co., Ltd. (Beijing, China). Antibiotic-free medium in six-well plates was used to seed B-CPAP cells as 2.5 × 10^5^ cells/well. si-SESN2 or si-NC (9 μL) and lipofectamine 3,000 reagent was diluted in the same volume of Opti-MEM. Then mix two liquids, incubate for 10 min, and add dropwise to the plate. And the culture was continued until 48 h. Cells were then harvested 24 h after treatment with calycosin (100 μM) or control (0 μM).

### qRT-PCR

Total RNA from clinical sample tissues (triturated with a homogenizer on ice)/cell lysates were extracted using RNA TRIzol reagent (Invitrogen, United States) and reverse transcribed using the PrimeScript™ RT Kit (TaKaRa Dalian, China) as instructed and amplified using TB Green ® Premix Ex Taq™ II (#RR820A, TaKaRa). Relative expression levels of mRNA were determined using the 2^−ΔΔCt^ formula, and results were normalized with GAPDH. The primers were from the sangon bioech company (Jinan, China), and the sequences are shown in Table 1.

**TABLE 1:** The primers used in the study.

Gene	Primer sequence (5’→3′)	
SESN2	F: CCT​CTG​GGC​GAG​TAG​ACA​AC	R: GGA​GCC​TAC​CAG​GTA​AGA​ACA
Bax	F: CCC​GAG​AGG​TCT​TTT​TCC​GAG	R: CCA​GCC​CAT​GAT​GGT​TCT​GAT
Bcl-2	F: GGT​GGG​GTC​ATG​TGT​GTG​G	R: CGG​TTC​AGG​TAC​TCA​GTC​ATC​C
GAPDH	F: GGA​GCG​AGA​TCC​CTC​CAA​AAT	R: GGC​TGT​TGT​CAT​ACT​TCT​CAT​GG

### Western blot

Harvested protein from clinical sample tissue (grind with a homogenizer on ice)/cell by using the whole protein extraction kit (Keygen, China). Then sodium dodecyl sulfate-polyacrylamide gel electrophoresis was performed and transferred to a polyvinylidene fluoride membrane. Results were quantified by ImageJ software (NIH, United States) after being incubated with the primary antibodies overnight. p-AMPK, AMPK, p-mTOR, mTOR, p-P70S6K, and P70S6K antibodies were from Cell Signaling Technology (Massachusetts, United States), LC3 II/I, SESN2, GAPDH and β-actin were from proteintech (State of New Jersey, United States).

### Whole transcriptome microarray bioinformatics analysis

The B-CPAP cells were grown with calycosin (0.100 μM) for 24 h and then sent to GENE Matrix for sequencing (http://gcloud.taogene.com). The significantly different genes were screened with |Fold Change|≥1.5 and *p*-value<0.05 as the criteria, and parallel Gene Ontology (GO) and Kyoto Encyclopedia of Genes and Genomes (KEGG) analyses were performed.

### Clinical samples

The selected clinical samples (16 cases of PTC tissues, 16 cases of normal thyroid tissue) in this study were all from the Affiliated Hospital of Weifang Medical University and agreed upon by the patients. This study was approved by the Ethics Committee of Affiliated Hospital of Weifang Medical University.

### Statistical analysis

Statistical analyses were from GraphPad Prism 8.0 and expressed as means ± standard errors. Comparisons were assessed using unpaired Student’s t-tests/ANOVA according to different situations. Statistically significant at *p* < 0.05.

## Results

### Calycosin inhibited proliferation of B-CPAP cells

**FIGURE 1:**
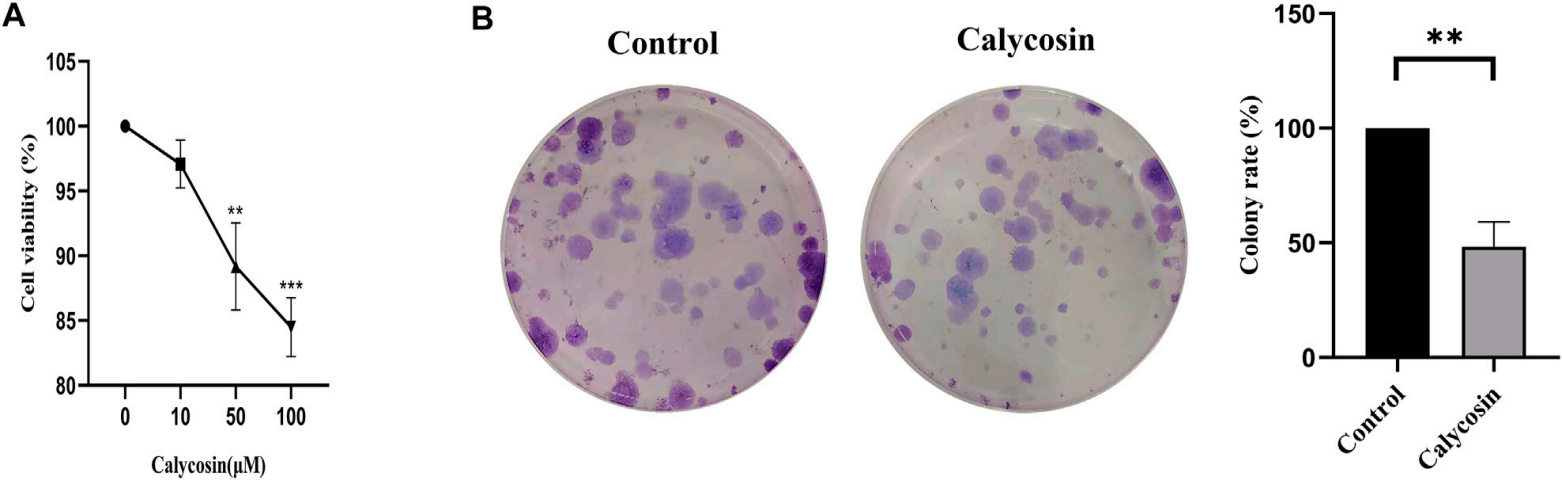
Calycosin inhibited the proliferation of B-CPAP cells. **(A)** CCK8 was used to measure the cell proliferation rate of BCPAP cells incubated with different concentrations of calycosin (0, 10, 50, 100 µM) for 24 h. **(B)** Colony formation assay was used to measure the number of clones formed in BCPAP cells incubated with calycosin (0, 100 µM) for 12 days. Results are representative of three independent experiments. *p < 0.05, **p < 0.01,***p < 0.001.

To determine whether calycosin has the anti-proliferative effects, the B-CPAP cells grown with calycosin was determined by CCK8 assay. With the increased calycosin concentration, cells' proliferation ability was gradually suppressed (Figure 1A). Afterward, we used a soft agar assay to analyze the effect of calycosin on B-CPAP cell-independent growth. The cell colonies formed by B-CPAP treated with calycosin (100 μM) were significantly less than that of the untreated cells after 12 days of culture, indicating that calycosin can inhibit B-CPAP cell proliferation (Figure 1B).

### Calycosin inhibited migration and invasion of B-CPAP cells

**FIGURE 2:**
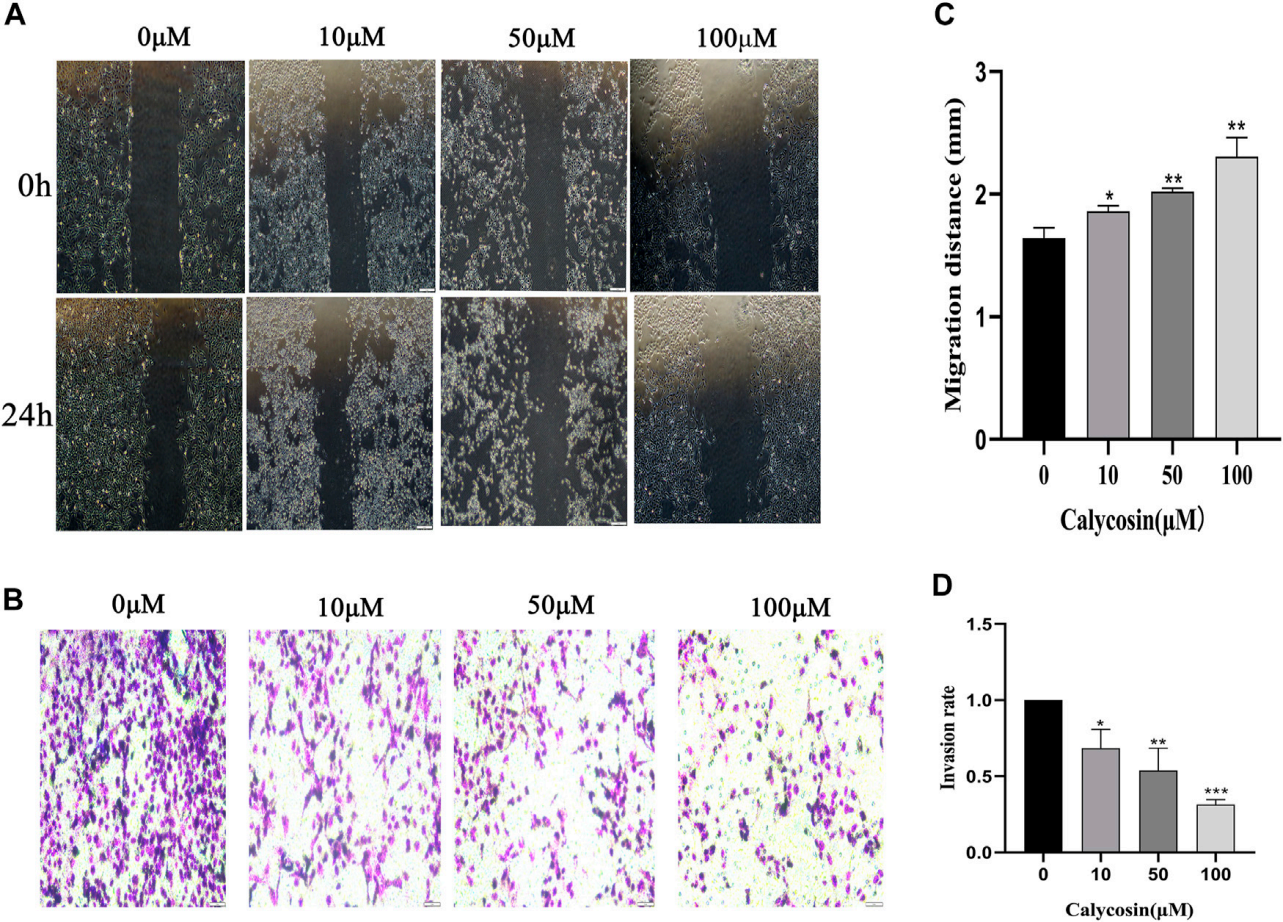
Calycosin inhibited the migration and invasion of B-CPAP cells. **(A)** Wound healing assay results in control or calycosin-treated B-CPAP cells at 0 and 24 h. **(C)** The distance between wound edges was measured. **(B)** Transwell invasion assay results showed the invasiveness of B-CPAP cells treated with calycosin. **(D)** The number of invasion cells was counted with ImageJ software. Results are representative of three independent experiments. *p < 0.05, **p < 0.01,***p < 0.001.

To analyze the effect of calycosin on cell migration and invasion, we further performed wound healing (Figure 2A) and transwell experiments (Figure 2B). With the increasing concentration of calycosin, the wound healing width after 24 h was higher (Figure 2C), and the cell migration and invasion rate were lower (Figure 2D). These data suggested that calycosin significantly inhibited the migration and invasion ability of B-CPAP cells.

### Calycosin induced apoptosis of B-CPAP cells

**FIGURE 3:**
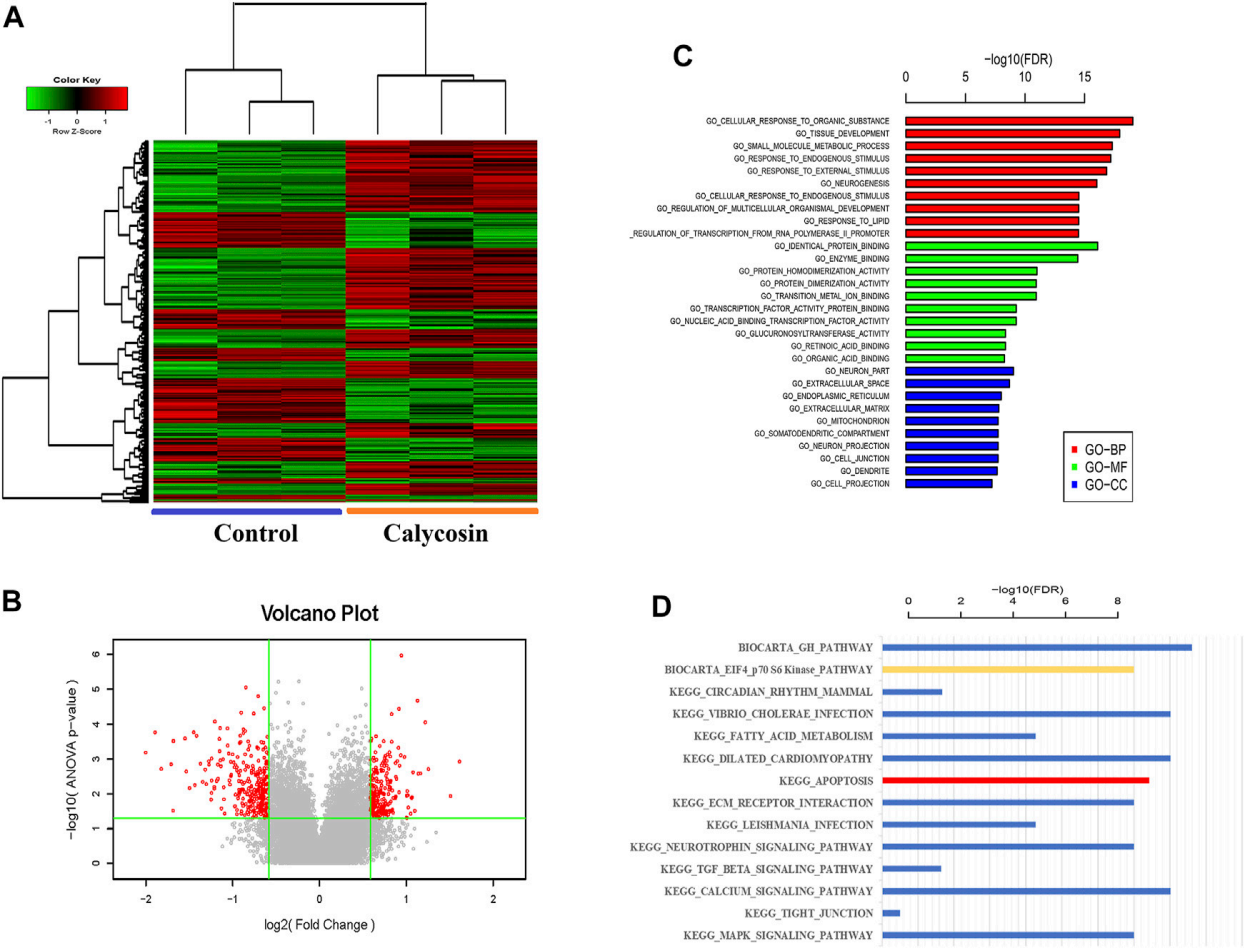
Whole Transcriptome Microarray Bioinformatics Analysis. **(A)** The DEGs of B-CPAP cells were treated with calycosin (0.100 µM) for 24 h. DEG screening criteria were: |Fold Change|≥1.5 and *p*-value<0.05. **(B)** Volcano plot: the red dots are points with |Fold Change|≥1.5 and *p*-value < 0.05 is a significant difference gene for standard screening, and the gray points are other genes with no significant difference. **(C)** The GO terms with the most significant DEG enrichment are listed. **(D)** The pathways with the most significant enrichment of KEGG in DEGs are listed.

**FIGURE 4:**
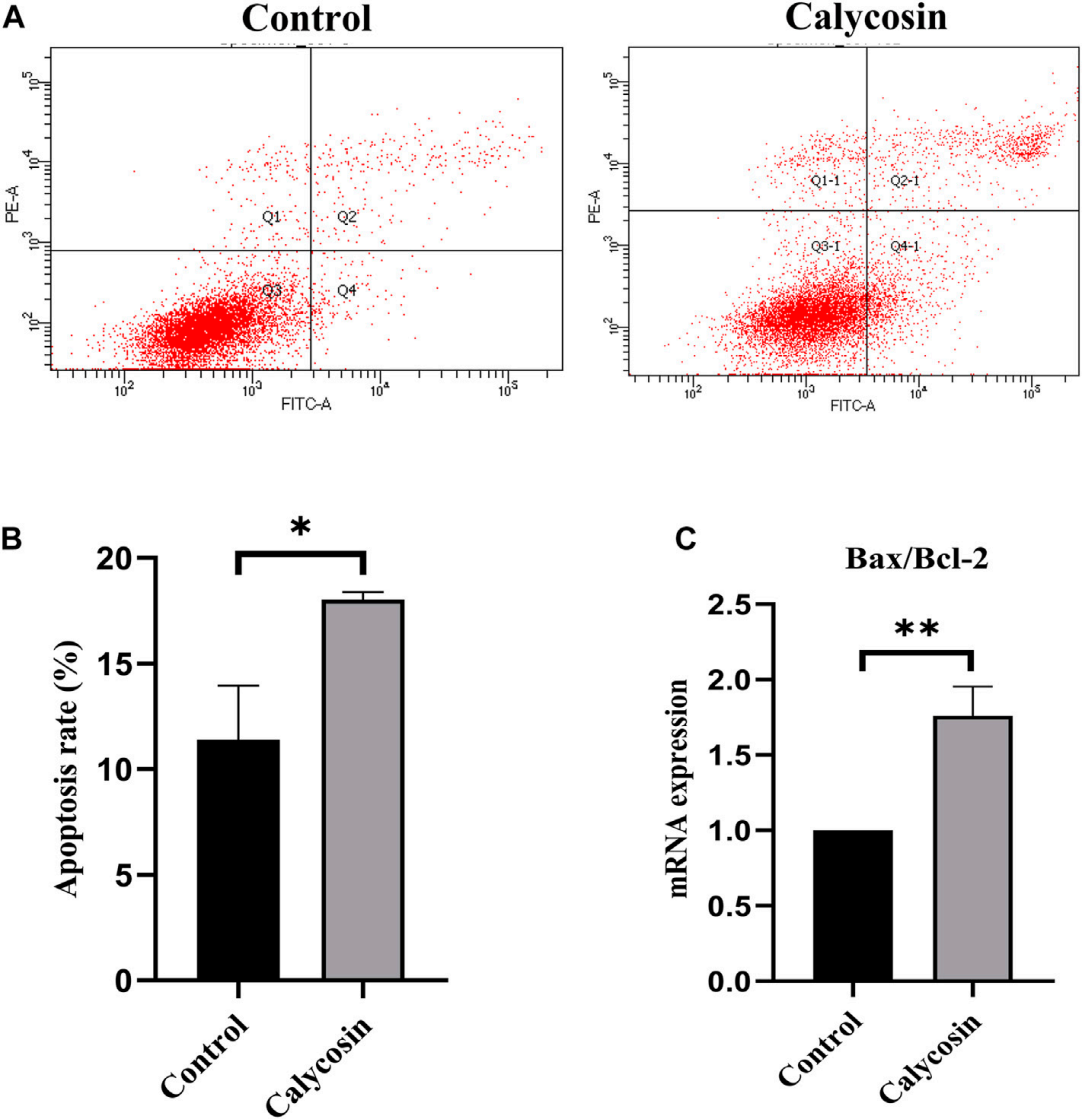
Calycosin promoted B-CPAP cell apoptosis. **(A)** B-CPAP cells were treated with calycosin (0.100 µM) for 24 h, and cell apoptosis was measured by flow cytometry. **(B)** The results were analyzed to obtain the apoptosis rate of each group. **(C)** Bax/Bcl-2 mRNA levels in B-CPAP cells. The experiment was repeated three times. *p < 0.05,**p < 0.01.

To identify potential protective mechanisms of calycosin on B-CPAP cells, the cells were grown with calycosin (100 μM) and performed expression microarray sequencing. Differentially expressed genes (DEGs) were screened out between the two groups (524 genes were upregulated and 328 genes were downregulated) (Figures 3A,B), and GO and KEGG of DEGs were analyzed: Adjusted.P.Value < 0.01 (Figures 3C,D). The pathway enrichment suggested that the role of calycosin in B-CPAP is closely related to apoptosis-related genes. To verify this result, we performed flow cytometry analysis (Figure 4A). The apoptosis rate of BCPAP cells treated with calycosin increased (Figure 4B) compared to control group. The relative expression of Bax/Bcl-2 in the calycosin group was higher than in untreated cells (Figure 4C). These results suggested that the calycosin treatment promoted the apoptosis of B-CPAP cells.

### Calycosin induced autophagy in B-CPAP cells

**FIGURE 5:**
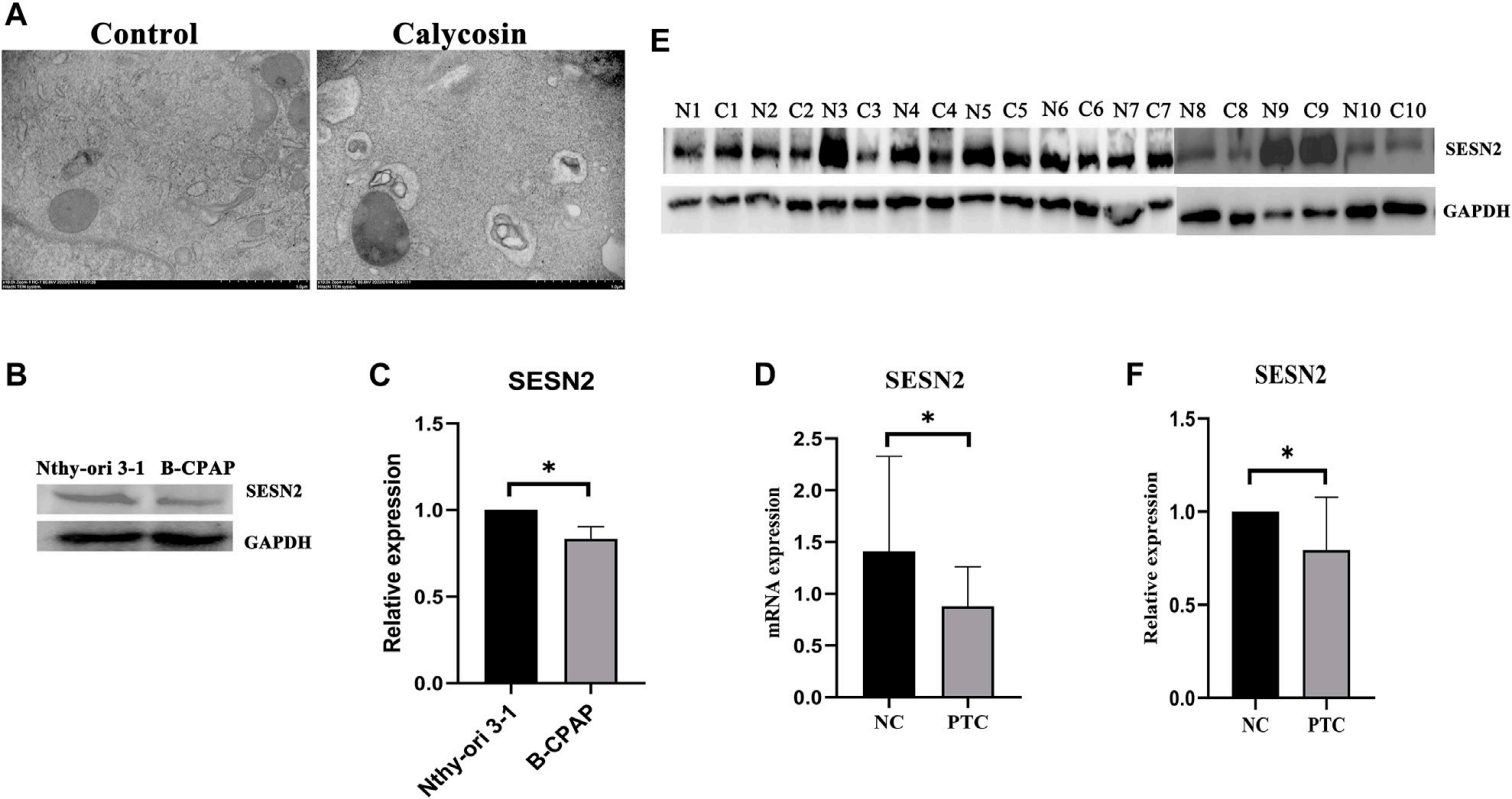
Calycosin induced B-CPAP cell autophagy **(A)** B-CPAP cells were treated with calycosin (100 µM) or control group for 24 h, washed with PBS, fixed with 2% glutaraldehyde, and imaged by transmission electron microscope to observe changes in cells. **(B,C)** SESN2 protein expression in Nthy-ori 3-1 and B-CPAP cells. **(D)** SESN2 mRNA expression in PTC tissue and human normal thyroid tissue. **(E,F)** SESN2 protein in human PTC tissue **(C)** and normal thyroid tissue (N). *p < 0.05.

The pathways enriched by the microarray sequencing results also showed that calycosin-treated B-APAP cells were associated with p70S6 Kinase (Figure 3D), a downstream target molecule of mTOR, which is a negative regulator of autophagy. To verify whether calycosin is related to autophagy, we observed cells by transmission electron microscopy and found an increase in autophagosomes in calycosin treatment group (Figure 5A). SESN2 is reported to activate autophagy *via* the AMPK/mTOR pathway in cancers. To investigate whether the antitumor effect of calycosin in PTC is also related to SESN2, we performed qRT-PCR and western blot experiments to verify the expression of SESN2 in cells and clinical sample tissue. We found that SESN2 in normal thyroid cells and tissues was higher than in B-CPAP cells (Figures 5B,C) and human PTC tissues (Figures 5D–F). The results indicated that calycosin promotes autophagy in B-CPAP cells and may be associated with SESN2 gene.

### Calycosin induced autophagy and apoptosis through the SESN2-AMPK-mTOR pathway

**FIGURE 6:**
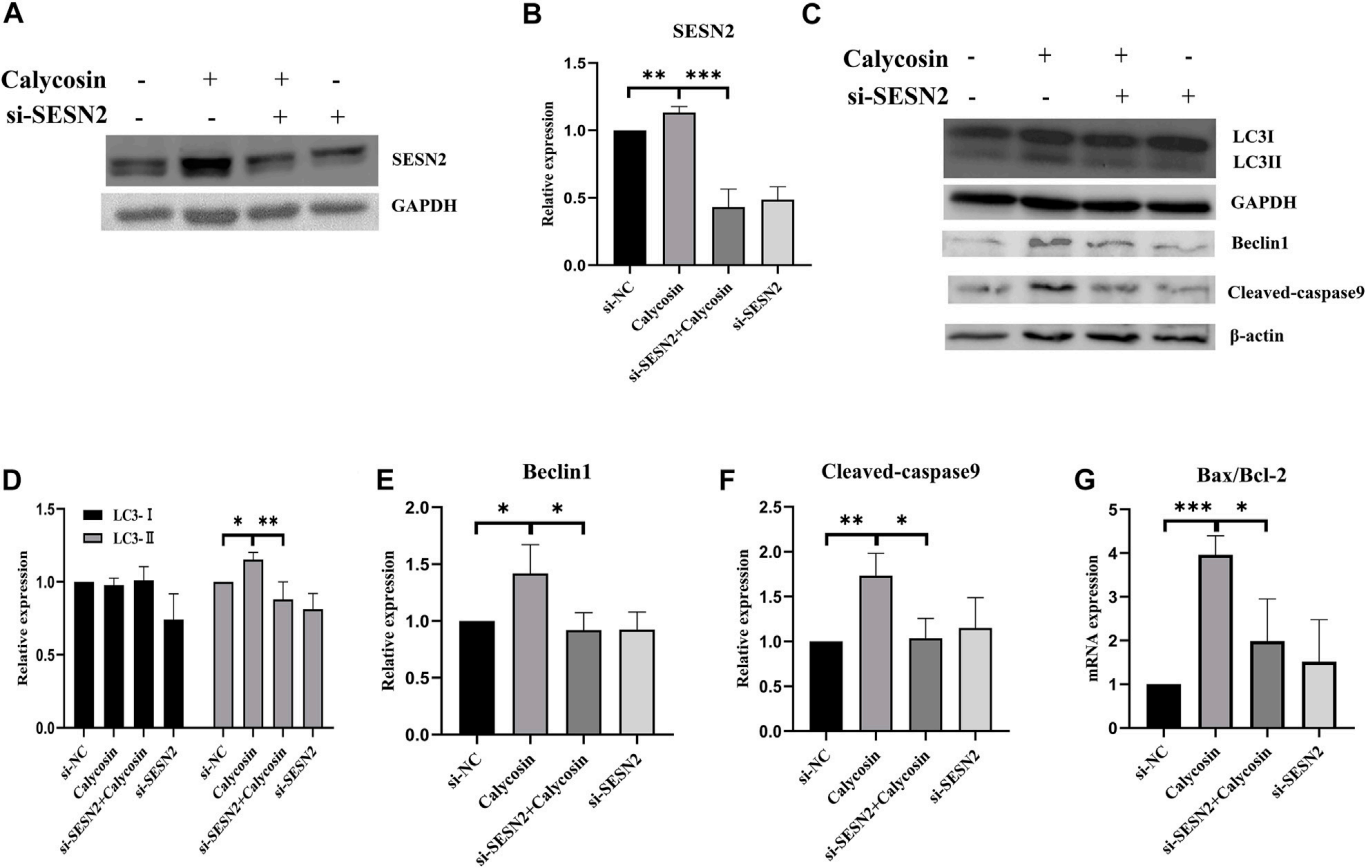
Calycosin induced apoptosis of B-CPAP cell and promoted autophagy related to SESN2. **(A,B)** The expression of SESN2 protein in B-CPAP cells treated with si-NC, si-SESN2 and/or calycosin (100 µM). **(C–F)** LC3Ⅰ/Ⅱ, Beclin1, Cleaved-caspase 9 proteins expression in B-CPAP cells treated with si-NC, si-SESN2 and/or calycosin (100 µM). **(G)** qRT-PCR analysis showing relative levels of Bax/Bcl-2 transcripts in si-NC, si-SESN2 and/or calycosin (100 µM)-treated B-CPAP cells. Results are representative of three independent experiments. *p < 0.05, **p < 0.01,***p < 0.001.

**FIGURE 7:**
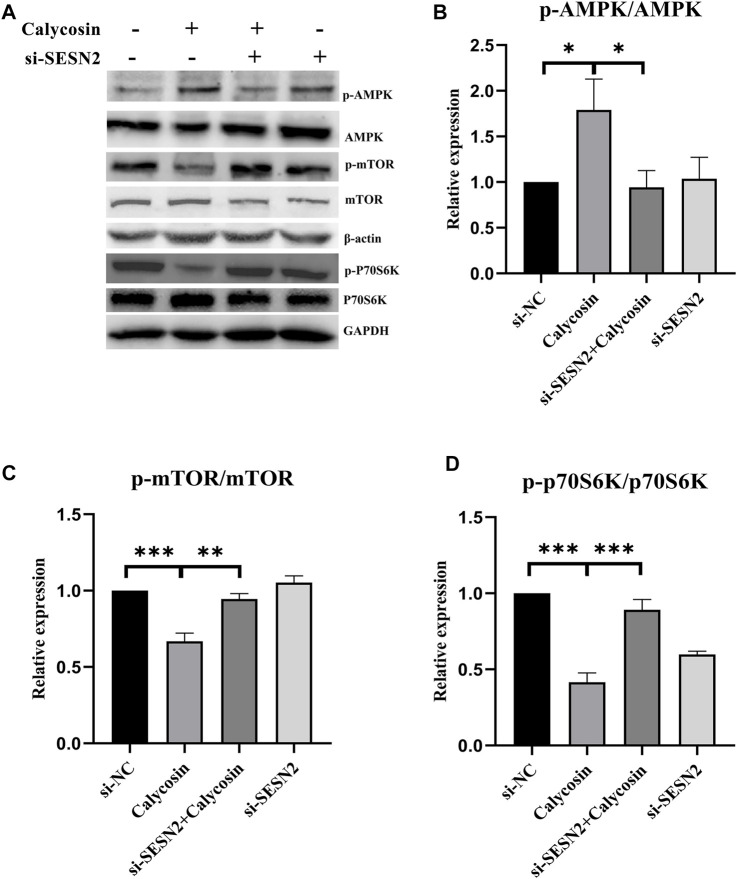
The pharmacological action of calycosin was through the SESN2-AMPK-mTOR pathway. The levels of p-AMPK/AMPK **(A,B)**, p-mTOR/mTOR **(A,C)** and p-p70S6K/p70S6K **(A,D)** proteins in si-NC, si-SESN2/calycosin (100 μM)-treated B-CPAP cells. Results are representative of three independent experiments. *p < 0.05, **p < 0.01, ***p < 0.001.

To further explore whether SESN2 was related to the protective effects of calycosin on autophagy and apoptosis, cells were transfected with siRNA targeting SESN2 (si-SESN2). We found that the protein expression of SESN2 was significantly increased after calycosin application (Figures 6A,B), which proved that the role of calycosin was indeed related to SESN2. The autophagic proteins (LC3II/I, Beclin1) and apoptosis markers (Bax/Bcl-2, Cleaved caspase-9) in the calycosin-treated cells did not increase after the SESN2-siRNA transfection (Figures 6C–G). These indicate that calycosin enhanced apoptosis and autophagy through SESN2 activation. Afterward, we continued to explore the SESN2-mediated downstream pathway. Calycosin significantly increased p-AMPK levels and inhibited p-mTOR and p-p70S6 kinase levels (Figure 7). These alterations were reversed when silencing SESN2, indicating that the antitumor effect of calycosin in B-CPAP cells was mediated by the SESN2/AMPK/mTOR pathway.

## Discussion

In this study, we found that calycosin reduced cell proliferation, inhibited migration and invasion, and promoted apoptosis of B-CPAP cells. This protective effect was associated with increased autophagy and activation of SESN2/AMPK/mTOR signaling pathway. Calycosin is a phytohormone with a molecular structure similar to mammalian estrogens and exerts biological activities, including anti-inflammatory activity, antioxidant activity, anti-osteoporosis activity, and anti-obese activity (Han et al., 2018; Deng et al., 2021). Several studies have demonstrated that calycosin has potential anti-metastatic effects on various tumors with low toxicity (Chen et al., 2011). However, until now, the role of calycosin in the PTC has never been studied. In this study, we examined the effect of calycosin on the growth of B-CPAP cells. Both the CCK8 assay and colony formation assay confirmed the inhibitory effect of calycosin on B-CPAP cells with increased concentration. Further transwell and wound healing experiments confirmed that calycosin could inhibit the proliferation, migration and invasion of B-CPAP cells. These findings proved that calycosin significantly repressed cell proliferation, induced apoptosis, and suppressed cell invasion in human PTC cells.

The regulation of autophagy is a complex process involving several mechanisms (Li et al., 2020). The role of autophagy in tumors is twofold, including inhibiting tumor initiation and supporting tumor progression (Levy et al., 2017). Previous studies confirmed that activation of autophagy is beneficial to the treatment of PTC. In this study, the autophagy-related proteins in the calycosin group were significantly increased. And autophagosomes in B-CPAP cells after calycosin application was more than that in untreated control cells by transmission electron microscopy. The results confirmed that calycosin indeed activated autophagy in B-CPAP cells.

Different from autophagy, apoptosis is an active and orderly cellular process. Malignant cells reduce apoptosis to achieve tumor growth in various ways; thus, drugs promoting apoptosis can inhibit tumor progression. Autophagy can directly activate apoptosis upstream or promote cell survival and antagonize apoptosis. Previously, calycosin was reported to activate autophagy in colorectal cancer cells HT29 and induce cell death (El-Kott et al., 2019). Thus, we analyzed the relationship between autophagy and apoptosis in calycosin-treated B-CPAP cells. The pathways enriched by the microarray sequencing showed that calycosin-treated B-APAP cells were associated with apoptosis. Bax/Bcl-2 ratio was increased in B-CPAP cells after calycosin application. Further studies showed that the cleaved caspase-9 in the calycosin-treated cells was elevated compared to control. Moreover, flow cytometry verified that the apoptosis rate of B-CPAP cells treated with calycosin was higher than control. These results support that calycosin can activate autophagy and apoptosis of B-CPAP cells.

The initiation of autophagy is due to the induction of autophagy-related gene complexes and inhibition of the activity of mTORC1 (Kim and Guan, 2015). AMPK can inhibit cell growth by inhibiting mTOR/P70S6K signaling, thereby exerting anti-tumor effects (Egan et al., 2011). However, AMPK activator/mTOR inhibitor can effectively inhibit various thyroid cancer cell lines growth (Plews et al., 2015). SESN2 could regulate autophagy and apoptosis mainly by activating AMPK and inhibiting the mTOR/P70S6K pathway. Pathways enriched by microarray sequencing results also showed that calycosin-treated B-APAP cells were associated with p70S6Kinase. To investigate whether the antitumor effect of calycosin in PTC is also related to SESN2, we performed qRT-PCR and western blot experiments to verify the expression of SESN2 in PTC. The results showed that SESN2 was higher in PTC and B-CPAP than in normal thyroid tissue/cells, and also higher in which calycosin (100 μM) was applied than in untreated B-CPAP. After silencing SESN2, we found that the Bax/Bcl-2 ratio did not increase, and the LC3, Beclin1, and Cleaved caspase-9 did not change significantly. These indicate that calycosin did not affect activating autophagy/promoting apoptosis in B-CPAP cells after silencing SESN2, indicating that SESN2 played a role in promoting autophagy or apoptosis. Thus, we confirmed the role of calycosin in autophagy and apoptosis in B-CPAP was consistent. We then studied whether the antitumor effects of calycosin were related to the SESN2/AMPK/mTOR signaling pathway. We found that calycosin increased AMPK phosphorylation while decreasing p-mTOR and p-p70S6K protein. After SESN2 silencing, these proteins were not significantly changed, indicating that the apoptosis and autophagy of calycosin in B-CPAP cells were regulated through the SESN2/AMPK/mTOR signaling pathway.

This study also has some limitations. This study mainly focuses on the research in B-CPAP cells and small clinical samples; it has not been validated in multiple tumor cell lines, nor has it been validated for other types of thyroid cancer and *in vivo* experiments. More importantly, there is no further drug application and research on animal and clinical samples. In the next step, we will further conduct related experiments to deeply study the antitumor mechanism of calycosin on PTC, which is brought novel insights into the pathogenesis of PTC and may lead to new avenues for treatment.

## Conclusion

In conclusion, we found that calycosin can restrain B-CPAP cell proliferation, migration and invasion. Calycosin promoted apoptosis and autophagy *via* the SESN2/AMPK/mTOR pathway. Our study has revealed the anti-tumor pharmacological effects and the mechanism of calycosin on treatment of PTC. This supplies valuable rationale for clinical use of calycosin in treating PTC.

## Data Availability

The original contributions presented in the study are included in the article/Supplementary Material, further inquiries can be directed to the corresponding authors.
